# Biological Characteristics, Hazard Patterns, and Control Measures of *Aegilops tauschii*, the Most Harmful Weed in Chinese Wheat Fields

**DOI:** 10.3390/plants14111607

**Published:** 2025-05-24

**Authors:** Yaling Geng, Chencan Wang, Jiangwei Han, Yiyun Ban, Zongran Su, Linghui Wang, Jing Xu, Libing Yuan

**Affiliations:** 1 Plant Protection Institute, Hebei Academy of Agriculture and Forestry Sciences, Baoding 071001, China; gengyaling2006@163.com (Y.G.); w3387134833@163.com (C.W.); 15830902389@163.com (Y.B.); 15097791534su@sina.com (Z.S.); wlh15932127411@163.com (L.W.); 15350564723@163.com (J.X.); 2 Key Laboratory of Integrated Pest Management on Crops in Northern Region of North China, Ministry of Agriculture and Rural Affairs, P. R. China, Baoding 071001, China; 3 IPM Innovation Center of Hebei Province, Baoding 071001, China; 4 International Science and Technology Joint Research Center on IPM of Hebei Province, Baoding 071001, China; 5 Shijiazhuang Seed Management Station, Shijiazhuang 050021, China; hanjiangwei83@126.com

**Keywords:** *Aegilops tauschii* Coss., weed, herbicide, emergence characteristics, agricultural-control measures, yield loss model, population-development dynamics

## Abstract

The control of *A. tauschii* is critical to ensuring food security. This study investigated a range of different aspects of the biology of *A. tauschii*, including its emergence characteristics, population development dynamics, and its impact on wheat yield. Moreover, the efficacy of different herbicides and cultural control measures for managing *A. tauschii* was explored. Through laboratory cultivation and statistical analysis of the emergence rate of *A. tauschii*, it was found that its emergence rate significantly increased when temperatures ranged from 10 °C to 20 °C and the environmental osmotic potential fell between −0.1 MPa and −0.5 MPa—conditions similar to those found in wheat fields. Additionally, by recording the emergence rates at different depths, *A. tauschii* emergence was found to occur optimally at a sowing depth of 1–5 cm, which aligns with the shallow rotary tillage currently employed in wheat production. The weed was also found to be tolerant to weakly acidic and alkaline environments, while also presenting with moderate salt tolerance. Through field experiments, it was found that, upon spreading to new areas, *A. tauschii* rapidly expanded its population size. While its impact on wheat yield was relatively mild during the early stages of growth, it escalated to severe outbreaks with the passage of time. Field experiments were conducted to test the efficacy of five herbicides on weed control. The analysis indicated that Mesosulfuron-methyl was the only effective herbicide in controlling *A. tauschii*. Adopting three two-year-three-crop rotation patterns reduced the density of *A. tauschii* from 186 stems/m^2^ to 11–15 stems/m^2^. Watering-induced emergence also proved effective. The most effective watering was performed 15 days before sowing. Deep plowing was another effective measure. The deeper the plowing, the lower the emergence of *A. tauschii*. Delayed sowing time resulted in the additional suppression of the emergence of *A. tauschii*.

## 1. Introduction

Wheat is the second largest stable crop in the world, and maintaining high yields of this grain is thus vital to global food security [1,2], while also providing a strong basis for social stability and economic development. Weed control is a key consideration in the context of wheat production, as effective weed control is vital to achieving superior crop yields and quality [3]. Recent years have been marked by a rise in the harm caused by *Aegilops tauschii* (*A. tauschii*) as a weed in wheat fields, posing a serious threat to reliable wheat production.

*A. tauschii* Coss. (2 n = 2 x = 14, DD) is a diploid self-pollinating plant in the *Aegilops* genus that grows extensively through the Mediterranean coast of West Asia, the Middle East, Southeastern Europe, and Northern Africa. It is primarily distributed in regions that produce large quantities of wheat situated between 30 and 45 degrees north latitude. Like in the case of many other weeds, such as *Ambrosia artemisiifolia* [4], global climate change has contributed to the overall expansion of the potential distribution range of *A. tauschii* [5]. Both wild natural populations and weed-type populations of *A. tauschii* have been documented. The former comprises a natural community distributed throughout natural habitats with high levels of genetic diversity [6,7], and it has even been proposed as a valuable genetic resource for use in the context of wheat breeding [8,9,10,11,12]. The latter, however, has established itself as a weed found in wheat fields throughout China, Iran, Afghanistan, and Pakistan. While the prolonged use of tribenuron-methyl and other herbicides in China has led to the control of the once-dominant weed species, *A. tauschii* and other gramineous species have taken their place as the most common weeds found growing in wheat fields [13]. Wheat and *A. tauschii* grow in a similar niche [14], with the latter exhibiting similar growth dynamics, emergence timing, and morphological features to those of wheat such that it competes with wheat for access to resources including water, light, and fertilizer. After harvesting, *A. tauschii* spikes can easily become intermixed with wheat grains and their removal is challenging, thereby having adverse direct or indirect effects on wheat yield and quality [15,16,17,18,19,20]. Wheat yields in areas with average levels of *A. tauschii* growth can reportedly be reduced by 12–15%, with reductions of up to 20–38% in areas with particularly high levels of weed growth and >50% yield reductions in severely infested areas. This issue is further compounded by the strong tillering ability of *A. tauschii* [21], together with its propensity for rapid propagation, ease of dissemination, and status as an alternative host for wheat stripe rust [11]. Given the challenges associated with manual weeding-based removal of this species and the insufficient availability of effective herbicides, *A. tauschii* is widely regarded as one of the most economically harmful and difficult-to-control weeds encountered in wheat fields [22]. Strategies for effectively controlling the spread of *A. tauschii* and associated harm are a major focus of ongoing research. Most farmers elect to spray herbicides on their wheat fields as a weed control strategy [23]. However, only one compound capable of controlling *A. tauschii* in wheat fields without also harming wheat—mesosulfuron-methyl—has been identified to date, and it is readily absorbed through the stems and leaves of these weeds [24]. As it inhibits acetolactate synthase activity in plants by interacting with a single site in this enzyme, weeds can readily develop resistance to this herbicide through the mutation of a single gene locus. Other agricultural control strategies beyond chemical control have also been employed to control weeds. Prior to sowing, for instance, the leveling of farmland is important to remove weed seeds, while also eliminating those weeds present along field edges to prevent the introduction of their seeds during sowing. Employing agricultural methods for weed control can not only protect the ecological environment, but also achieve effective weed management and enhance overall farming practices. One such method is deep turning rotation [25]. By burying weed seeds deep into the soil, agricultural workers can significantly reduce the germination and emergence of weeds [26,27]. Furthermore, crop rotation can alter the environment through differences in crop management practices, disrupting the favorable growth conditions for weeds and thereby achieving effective control. Therefore, adopting an integrated management strategy that prioritizes agronomical measures and supplements them with chemical control is crucial for improving wheat yield, mitigating the impact of *A. tauschii* on wheat growth, and promoting sustainable agricultural development.

Given that the range and severity of *A. tauschii* infestations continue to increase, this study sought to explore and implement the development of a more robust understanding of the weed’s occurrence and infestation through the examination of trends in population growth, its effects on wheat, and the characteristics that define its emergence. The associations between population growth and agronomical practices relevant to this weed species were also explored at length, with the ultimate goal of disrupting *A. tauschii* growth habitats as a means of reducing or eliminating its occurrence. This study has laid a theoretical foundation for establishing a comprehensive weed control system for wheat, which is significant for improving the effectiveness of *A. tauschii* control, enhancing wheat production, and ensuring safety.

## 2. Results

### 2.1. Characterization of A. tauschii Emergence

#### 2.1.1. Characterization of *A. tauschii* Seed Dormancy

**Figure 1:**
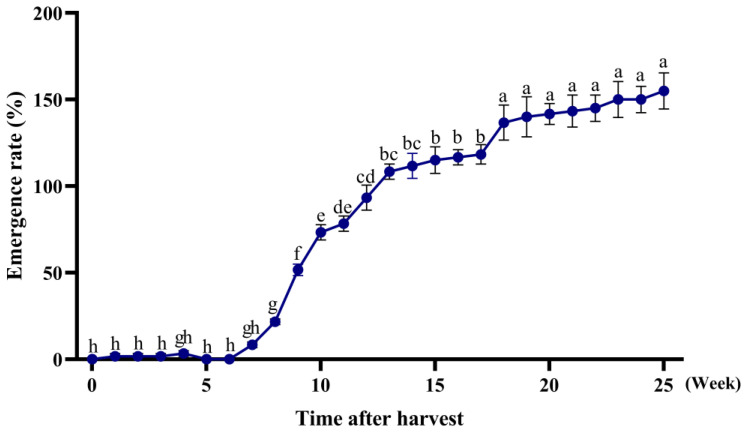
*A. tauschii* seedling emergence at different times post-maturity. Different lowercase letters on the line chart indicate significant differences in different weeks (*p* < 0.05).

The seedling emergence rates 14 days after sowing are presented in Figure 1 (F_2,50_ = 26.68, *p* < 0.0001). The results show that seed dormancy began to diminish in the seventh week after maturity. The emergence rates increased rapidly between the 7th and 13th week, more gradually from the 14th to 17th week, and largely plateaued after the 18th week.

#### 2.1.2. Effects of Soil Depths on *A. tauschii* Seedling Emergence

**Figure 2:**
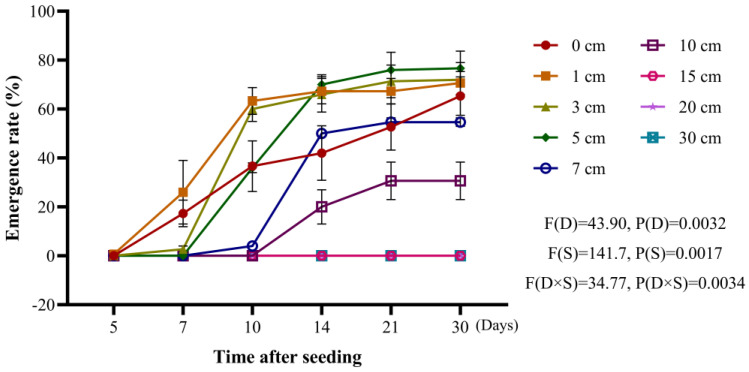
Effect of sowing depth on the seedling emergence rates of *A. tauschii*. In the statistical results, (D) and (S) represent the independent factors of day and soil depth, respectively.

The emergence rate of *A. tauschii* was highest at soil depths of 1 cm, 3 cm, and 5 cm, reaching approximately 75% within 30 days of sowing. However, the periods of peak emergence varied: 5–10 days for a 1 cm depth, 7–10 days for 3 cm, and 7–14 days for 5 cm. The emergence period for the seeds sown on the soil surface (0 cm) was prolonged despite an emergence rate of 65%. For depths of 7 cm and 10 cm, the emergence rate decreased progressively, while no emergence occurred at depths greater than 15 cm (Figure 2).

#### 2.1.3. Effects of Different Environmental Factors on *A. tauschii* Radicles and Seedling Emergence

**Figure 3:**
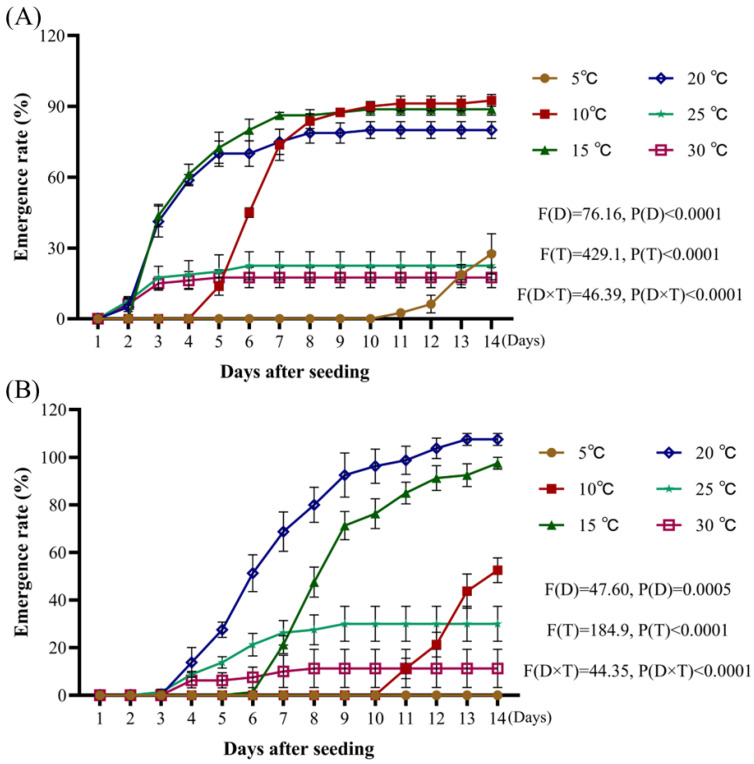
Effects of temperature on emergence rate of *A. tauschii* radicles (**A**) and seedlings (**B**). In the statistical results, (D) and (T) represent the independent factors of day and temperature, respectively.

When assessing temperature and using the radicle emergence rate as an index for analysis, the most favorable temperature for *A. tauschii* germination was 10 °C, where the emergence rate reached 92.5% within 14 days, with a peak germination period around 10 days after sowing. At 15 °C, the radicle emergence rate was slightly lower (87.5%) but peaked earlier at 7 days. At 20 °C, the emergence rate peaked on day 8 and reached 80% after 14 days. In contrast, emergence rates at 25 °C and 30 °C were much lower at approximately 20% after 14 days, despite an earlier germination onset. At 5 °C, germination was delayed and reached only 27.5% after 14 days (Figure 3A).

When assessing temperature and using the seedling emergence rate as an index for analysis, 20 °C was optimal, with an emergence rate of 107.5% after 14 days and a largely concentrated period of emergence. Note that this rate exceeded 100% because each spikelet contains 2–3 seeds, all of which may develop into a seedling. The second highest emergence rate was observed at 15 °C (97.5%), followed by 10 °C (52.5%). Seedlings at 25 °C and 30 °C emerged earlier (day 4) but had low emergence rates (up to 30%). At 5°C, no seedlings emerged within 14 days (Figure 3B).

**Figure 4:**
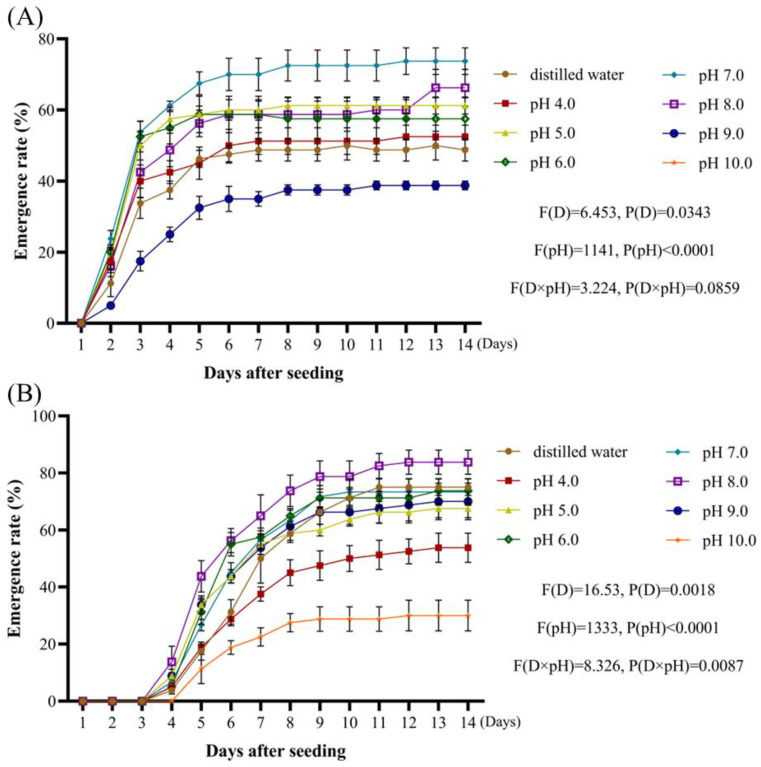
The effect of pH on the emergence rates of *A. tauschii* radicles (**A**) and seedlings (**B**). In the statistical results, (D) and (pH) represent the independent factors of day and pH value, respectively.

When assessing the effects of pH and using the radicle emergence rate as an index for analysis, radicle emergence was highest at pH 8.0, exceeding the control (distilled water). The emergence rates at pH 6.0, 7.0, and 9.0 were similar to the control, while those at pH 4.0, 5.0, and 10.0 were significantly lower, with pH 10.0 being the least favorable (Figure 4A). Seedling emergence followed a similar trend, with pH 8.0 showing the highest rate, surpassing the control. Emergence rates at pH 6.0 and 7.0 were comparable to the control, while those at pH 4.0, 5.0, 9.0, and 10.0 were lower, with pH 10.0 being the least conducive to emergence (Figure 4B). Under these different pH levels, peak radicle emergence tended to be observed around 6 days after sowing, with the highest seedling emergence rate around day 9 after sowing. Overall, *A. tauschii* exhibited tolerance to slightly acidic and alkaline conditions, with no significant differences in radicle emergence between pH 6.0 and 9.0 or in seedling emergence between pH 6.0 and 8.0.

**Figure 5:**
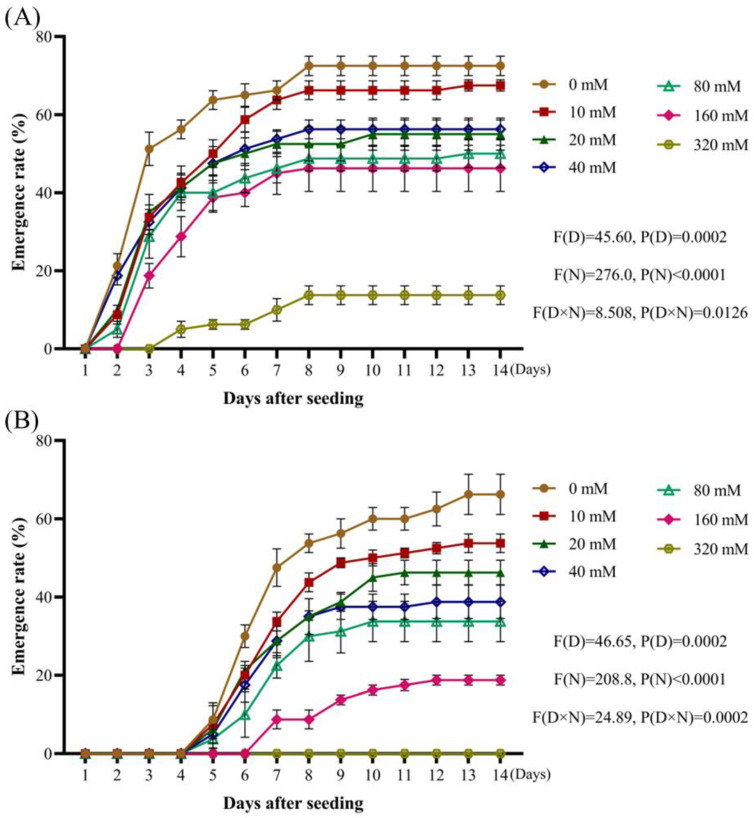
Effects of NaCl concentration on the emergence rates of *A. tauschii* radicles (**A**) and seedlings (**B**). In the statistical results, (D) and (N) represent the independent factors of day and NaCl concentration, respectively.

The radicle emergence rate of *A. tauschii* decreased as the NaCl concentration increased. Fourteen days after sowing, the radicle emergence rate was highest under the distilled water treatment, reaching 72.5%. At an NaCl concentration of 10 mmol/L, the radicle emergence rate decreased slightly by 5%, indicating a minimal impact. However, at NaCl concentrations ranging from 20 to 160 mmol/L, the radicle emergence rate declined by 16.25–26.25%, indicating a significant inhibitory effect. A concentration of 320 mmol/L severely inhibited radicle emergence, reducing the rate by 58.75% (Figure 5A). Similarly, *A. tauschii* seedling emergence gradually decreased with increasing NaCl concentrations. Fourteen days after sowing, compared to the distilled water treatment, NaCl concentrations of 10–160 mmol/L significantly inhibited seedling emergence, causing a reduction of 12.5–47.5% from the initial rate of 66.25%. At a concentration of 320 mmol/L, the seedling emergence rate was reduced to 0% (Figure 5B).

**Figure 6:**
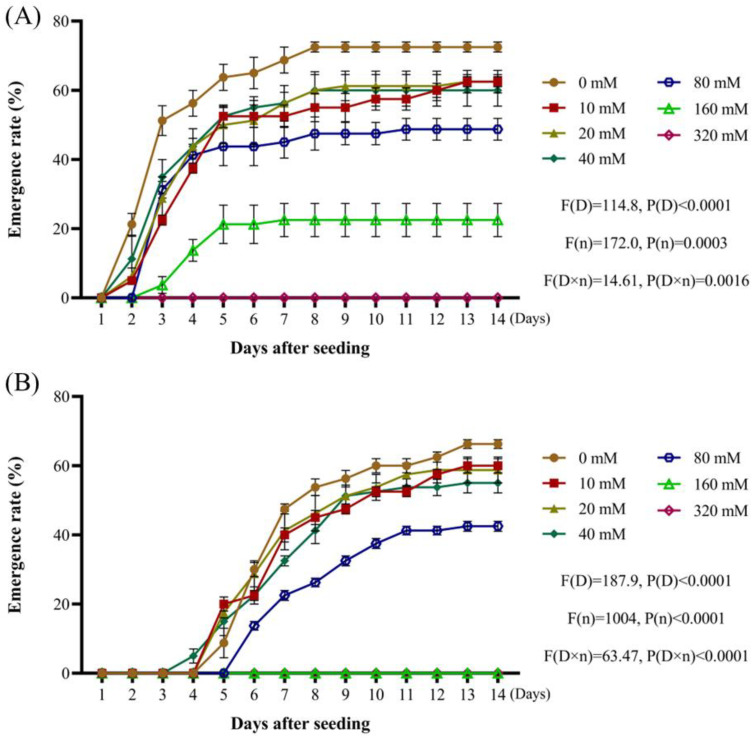
Effects of Na_2_SO_4_ concentration on the emergence rates of *A. tauschii* radicles (**A**) and seedlings (**B**). In the statistical results, (D) and (n) represent the independent factors of day and Na_2_SO_4_ concentration, respectively.

When the effects of Na_2_SO_4_ concentrations were evaluated, the radicle emergence rate of *A. tauschii* also decreased with increasing concentrations. Fourteen days after sowing, the radicle emergence rates dropped by 10–12.5% under Na_2_SO_4_ concentrations of 10–40 mmol/L compared to the distilled water treatment, representing a relatively minor impact. However, at an Na_2_SO_4_ concentration of 80 mmol/L, the radicle emergence rate declined significantly by 25%. At 160 mmol/L, the rate was reduced by 50%, and at 320 mmol/L, radicle emergence was completely inhibited (Figure 6A). With respect to seedling emergence, a similar trend was observed. Fourteen days after sowing, Na_2_SO_4_ concentrations of 10–40 mmol/L reduced the seedling emergence rate by 6.25–10% compared to distilled water, with this effect not being significant. At 80 mmol/L, the seedling emergence rate decreased by 23.75%, consistent with a substantial impact. At concentrations of 160 and 320 mmol/L, the seedling emergence rate dropped to 0% (Figure 6B). These findings demonstrate that both NaCl and Na_2_SO_4_ inhibit the emergence of *A. tauschii*, with higher concentrations causing more pronounced inhibition.

**Figure 7:**
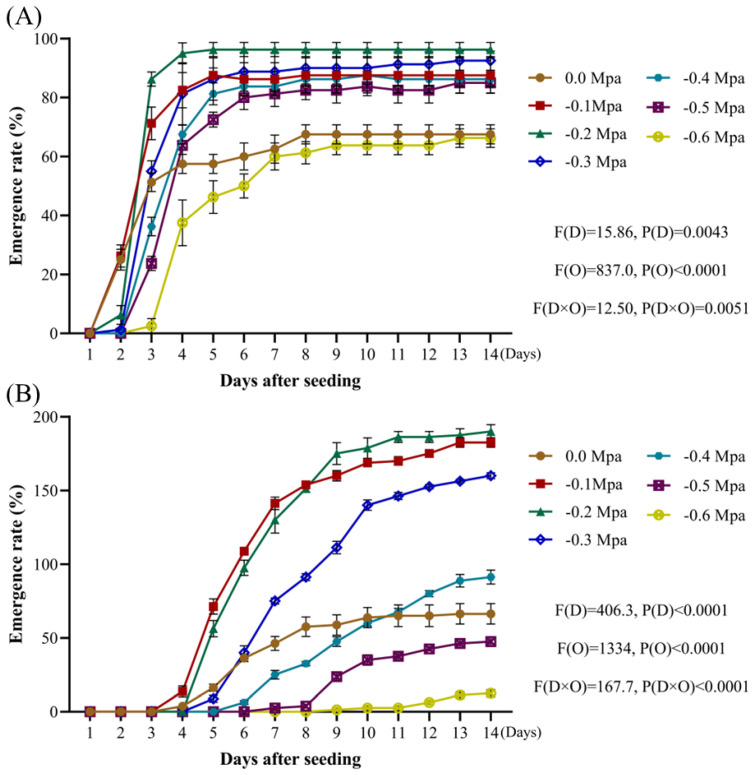
The impact of osmotic potential on the emergence rates of *A. tauschii* radicles (**A**) and seedlings (**B**). In the statistical results, (D) and (O) represent the independent factors of day and osmotic potential, respectively.

Osmotic potential also influenced the emergence rates of *A. tauschii*. With respect to radicle emergence, osmotic potentials between −0.1 and −0.5 MPa significantly increased the radicle emergence rate compared to distilled water. However, there was no significant difference between the osmotic potential of −0.6 MPa and the distilled water treatment (Figure 7A). In terms of seedling emergence, osmotic potentials between −0.1 and −0.3 MPa significantly improved the emergence rates. At an osmotic potential of −0.4 MPa, the seedling emergence rates were initially lower than those under the distilled water treatment from days 4–10 but they surpassed them by days 11–14. When the osmotic potential ranged from −0.5 to −0.6 MPa, the seedling emergence rates were markedly lower than those observed under the distilled water treatment (Figure 7B).

### 2.2. Characterizing the Dynamics of A. tauschii Population Development

**Figure 8:**
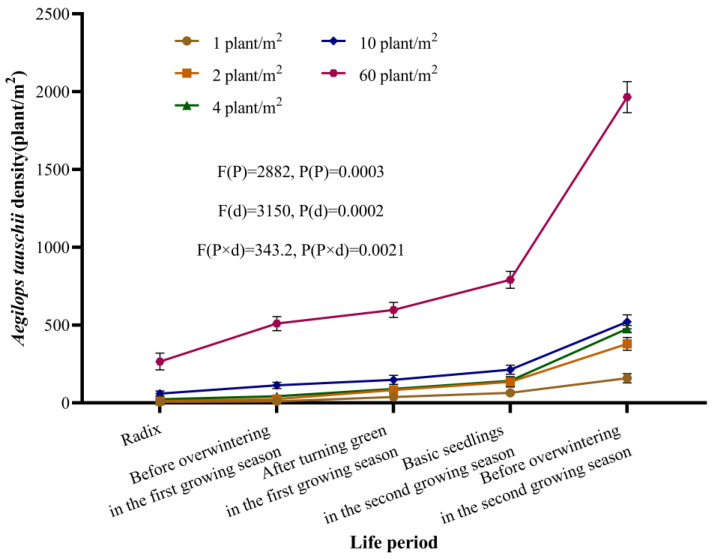
Development of *A. tauschii* populations with varied initial densities. In the statistical results, (P) and (d) represent the independent factors of life period and initial density of *A. tauschii*, respectively.

When the initial density of *A. tauschii* was 1–2 plants/m^2^, the population density increased by approximately 190-fold during the second growing season. With an initial density of 4–10 plants/m^2^, the population density increased by about 60-fold. At an initial density of 60 plants/m^2^, the population density increased 34-fold during the second growing season (Figure 8), highlighting the marked expansion of this weed species.

### 2.3. Yield Losses Caused by A. tauschii

**Figure 9:**
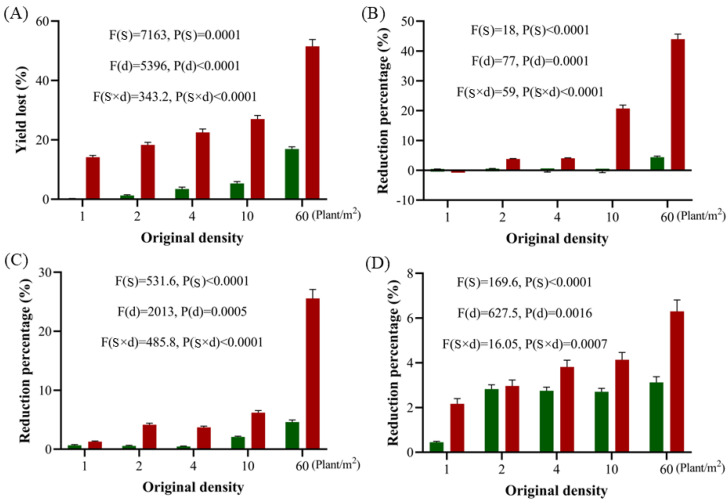
Effects of *A. tauschii* population density on wheat yield losses (**A**), panicle numbers (**B**), grain numbers per panicle (**C**), and 1000-grain weights (**D**). Green and red columns present the first and second growing season, respectively. In the statistical results, (S) and (d) represent the independent factors of growing season and initial density of *A. tauschii*, respectively.

The population density of *A. tauschii* had a direct effect on wheat yield. At densities of 1–10 plants/m^2^, wheat yield was reduced by 0.25–5.33% during the first growing season and by 14–27% during the second growing season as population density increased. At a density of 60 plants/m^2^, wheat yield decreased by 17% in the first growing season and by 52% in the second (Figure 9A). For an initial population density of 1–4 plants/m^2^, the reductions in the number of panicles, grains per panicle, and the 1000-grain weight across two growing seasons were less than 5%. However, at densities of 10–60 plants/m^2^, the first growing season showed less than 5% reductions in these yield components. In the second growing season, the panicle numbers decreased by 21–44% (Figure 9B), the grains per panicle decreased by 6–27% (Figure 9C), and the 1000-grain weight decreased by 4–6% (Figure 9D).

These results indicated that *A. tauschii* populations can establish and proliferate rapidly in wheat fields at densities of 1–60 plants/m^2^. Smaller initial densities were associated with faster proliferation rates. Based on the correlation between *A. tauschii* population density and wheat-yield loss, a yield loss model was developed using probability analysis with the following formula:

Y = 2.6589 + 0.8011 X.

where X is the logarithm of the density of *A. tauschii* (basic seedlings), and Y is the probability of the yield loss rate. The correlation coefficient for this formula was 0.9337, with a *p* value of 0.0002.

### 2.4. Evaluation of the Efficacy of Different A. Tauschii Control Measures

#### 2.4.1. Effect of Different Herbicides on *A. tauschii*

**Figure 10:**
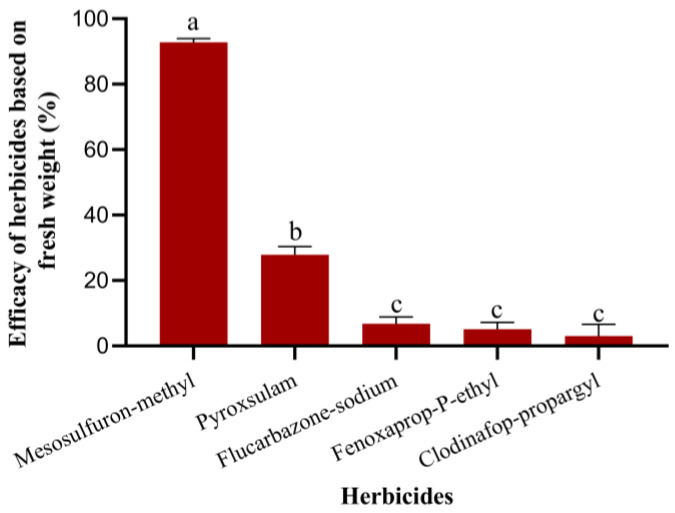
Assessment of the control performance of different herbicides on *A. tauschii* in wheat fields. Different lowercase letters on the column indicate significant differences after applying different herbicides (*p* < 0.05).

The resultant data results showed that mesosulfuron-methyl OD provided the best control, reducing the fresh weight of weeds by 93%. Pyroxsulam OD achieved a 28% reduction, while flucarbazone-sodium WG, fenoxaprop-p-ethyl EW, and clodinafop-propargyl WP reduced fresh weed weight by only 7%, 5%, and 3%, respectively. These results indicate that mesosulfuron-methyl is the most effective herbicide for controlling *A. tauschii* in wheat fields (Figure 10, F_4,15_ = 244.4, *p* < 0.0001).

#### 2.4.2. Effect of Different Cultural Control Methods on *A. tauschii*

**Figure 11:**
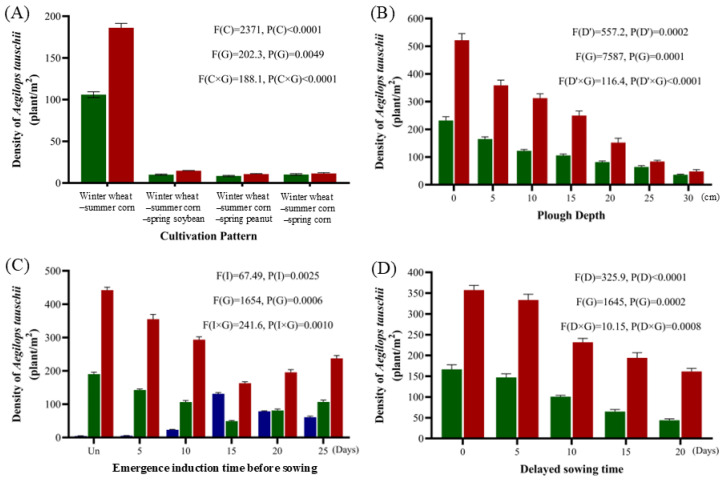
Effects of cropping systems (**A**), plowing depths (**B**), emergence induction (**C**). Un stands for non-induced emergence, delayed sowing (**D**) on the occurrence of *A. tauschii*. Blue, green and red columns present before sowing, before overwintering, and after spring regrowth, respectively. In the statistical results, (C) in (**A**) indicates the different crop rotation, (D’) in (**B**) indicates the plowing depth, (I) in (**C**) indicates the emergence induction time, (D) in (**D**) indicates the delayed sowing time, and (G) indicates the growth period, respectively.

In addition to chemical methods, agronomic practices such as crop rotation, deep plowing, inducing emergence before tillage, and delayed sowing were evaluated for their efficacy in controlling *A. tauschii*. The purpose of the field experiment was to compare the density changes of *A. tauschii* before wintering and after spring regrowth between three different two-year, three-cropping systems and the conventional winter wheat-summer corn double-cropping system. Under the conventional system, the density of *A. tauschii* was 106 plants/m^2^ before overwintering and 186 plants/m^2^ after spring regrowth. In contrast, the three-cropping systems significantly reduced weed density to 8–10 plants/m^2^ before overwintering and 11–15 plants/m^2^ after regrowth (Figure 11A).

The effect of plowing depth on *A. tauschii* density was also assessed. The results showed that deeper plowing significantly reduced weed density compared to untreated plots, with greater reductions observed at greater depths (Figure 11B).

Additionally, the effectiveness of watering-induced emergence on *A. tauschii* was evaluated. Inducing weed emergence 15 days before wheat sowing, followed by rotary tillage, resulted in the greatest reduction in *A. tauschii* density, with a 74% decrease before overwintering and a 63% decrease after spring regrowth compared to untreated plots (Figure 11C).

Delayed sowing was also studied, revealing that later sowing significantly reduced *A. tauschii* density. However, excessively delayed sowing negatively affected wheat growth and yield (Figure 11D).

## 3. Discussion

*Aegilops tauschii* Coss., a highly problematic and invasive weed species, poses increasingly significant challenges to modern agricultural systems worldwide [28]. This weed shares a substantial ecological niche overlap with wheat, leading to intense and direct competition for essential resources such as sunlight, nutrients, water, and physical growing space. The resulting competition drastically reduces both the yield and quality of wheat crops, thereby posing a serious threat to the stability, sustainability, and security of global wheat production systems [29]. In this study, we conducted a thorough analysis of the emergence characteristics, population dynamics, and impacts of *A. tauschii* on wheat yields. The findings presented herein provide a crucial foundation for comprehending the occurrence and damage patterns of this invasive species and for developing effective strategies to manage and mitigate its impact.

*A. tauschii* seeds mature in late May, following which their dormancy typically terminates after approximately 18 weeks, coinciding with early October. This timing aligns precisely with the sowing period of winter wheat, resulting in the simultaneous emergence of both *A. tauschii* and wheat plants. This synchronization solidifies the role of *A. tauschii* as a major and persistent weed in wheat fields. However, conducting experiments with seeds stored at room temperature does not provide an accurate representation of the actual dormancy patterns observed in the field. The results presented in this paper can be regarded as a simulation experiment and serve as a reference for understanding dormancy behavior under field conditions. In our future efforts, we aim to improve the storage conditions of the seeds to closely mimic those encountered in the field. The ability of weed seeds to emerge under varying environmental conditions is a critical determinant of their survival, colonization, and subsequent spread. Previous research has demonstrated that seedling emergence depends heavily on seed reserves, which are often insufficient to support germination when buried in deeper soil layers [30]. Our study found that the optimal depth for the emergence of *A. tauschii* seeds was within the range of 1–5 cm, where emergence rates were at their highest, and the seedlings exhibited greater uniformity. Conversely, deeper planting depths resulted in reduced emergence rates, likely due to nutrient depletion occurring before the coleoptile reached the soil surface or because of sustained seed dormancy at deeper levels. Zhao et al. [31] reported that ungerminated seeds located in deeper soil layers can establish a persistent seed bank, germinating only under favorable environmental conditions. This “self-rescue” mechanism effectively ensures the long-term survival of the weed species. It is highly plausible that *A. tauschii* exhibits similar behavior. Based on these observed emergence characteristics, deep plowing is recommended as an effective strategy to bury *A. tauschii* seeds deeper within the soil, thereby reducing their ability to emerge and ultimately lowering the density of *A. tauschii* in wheat fields.

Weed seeds that exhibit tolerance to a wide range of acid-base and salt stress conditions tend to demonstrate enhanced competitiveness under various environmental stressors [32,33,34]. By analyzing the rates of *A. tauschii* seed emergence as a function of pH in this study, these seeds were found to possess a remarkably broad pH tolerance range. This adaptability highlights their ecological plasticity, which is a key factor contributing to the rapid and widespread dissemination of *A. tauschii*. Increasing salt concentrations resulted in a gradual decline in both radicle emergence and overall seedling emergence rates. Compared to other weed species, *A. tauschii* seeds exhibit moderate salt tolerance. When the osmotic potential ranged between −0.4 MPa and −0.1 MPa, the seed emergence rates were significantly enhanced. However, at an osmotic potential of −0.5 MPa and −0.6 MPa, seedling emergence was inhibited, although there was no significant suppression of radicle sprouting, and it was even enhanced in some cases. These findings suggest that mild drought stress conditions can promote the emergence of *A. tauschii*, whereas severe drought stress inhibits germination. This ensures that *A. tauschii* seeds do not germinate prematurely during humid summer conditions or under excessively dry environments, thereby enhancing their capacity to compete effectively within wheat fields.

When *A. tauschii* invades new areas, its initial population size is often small. However, the species is capable of forming dense and extensive populations within a relatively short period if left unchecked. To date, no studies have comprehensively reported the population growth patterns or the dynamic changes in infestation capacity as *A. tauschii* populations expand. Our multi-year, continuous experiments demonstrated that *A. tauschii* can establish and rapidly develop dense populations in wheat fields at densities ranging from as low as 1 plant/m^2^ to as high as 60 plants/m^2^ if no control measures are implemented. Remarkably, even a single plant per square meter can lead to the establishment of a population due to the species’ robust colonization capabilities. Once colonized, these populations expand rapidly, with initial infestations often being relatively inconspicuous but becoming increasingly evident over time. During the first growing season, the impact of *A. tauschii* on wheat yield was observed to be relatively minor. However, by the second growing season, wheat yield reductions ranging from 14% to 52% were recorded. Biomass was identified as the most responsive trait in our analyses, reflecting the highly competitive nature of *A. tauschii* growth. As weed density increases, its inhibitory effects on wheat become increasingly pronounced, primarily through reductions in the number of effective wheat ears and the 1000-grain weight. Consequently, exhaustive efforts are essential to prevent the spread of *A. tauschii* into new areas. In regions where the weed is already present, it is imperative to implement control measures early in the growing season to curb population growth and minimize the sizes of established infestations.

Based on the observed relationship between *A. tauschii* density and wheat-yield loss, a yield loss prediction model was developed using probability value analysis. Given the high correlation coefficient of this model, it can serve as a reliable tool for farmers and agricultural researchers to better understand and predict wheat yield losses caused by *A. tauschii* infestations. Moreover, it offers valuable guidance for devising effective prevention and control strategies tailored to the specific infestation densities encountered in wheat fields.

The control of *A. tauschii* requires a multifaceted, prevention-oriented, and integrated strategy that combines chemical herbicides with agronomic measures to achieve optimal results. Chemical control is widely recognized as a cornerstone of weed management due to its rapid action and high effectiveness in suppressing weed populations. However, controlling *A. tauschii* using herbicides presents several challenges, including the limited availability of effective options and the need for specific application conditions to ensure efficacy. This study identified mesosulfuron-methyl as the only herbicide effective in managing *A. tauschii*. This chemical was shown to inhibit the growth of the weed, ultimately leading to plant death. Nevertheless, the limitations of chemical herbicides are becoming increasingly apparent as agricultural practices evolve. The prolonged and repetitive use of herbicides containing specific active ingredients has been shown to result in the development of herbicide resistance in weed species, thereby reducing the long-term effectiveness of these chemicals. Consequently, there is an urgent need for the adoption of more diversified and sustainable cultural control methods to complement chemical approaches. These methods should address the root causes of weed proliferation while mitigating the risk of resistance.

In conventional agricultural systems, extensive farming practices have frequently contributed to the exacerbation of *A. tauschii* infestations. In many regions, farmers utilize a two-crop rotation system involving winter wheat and summer corn. After the wheat harvest, corn is often sown directly into the remaining stubble without plowing, leaving a significant portion of *A. tauschii* seeds on the soil surface. This practice leads to a gradual accumulation of *A. tauschii* seeds over time. Crop rotation, a long-established principle of integrated pest management, offers an effective means of disrupting the growth and reproductive cycles of weeds by redistributing soil nutrients and introducing variability into the growing environment. This variability limits the ability of weeds to adapt and thrive, ultimately reducing their impact on crops [35,36]. The present study explored different farming systems and found that, in comparison to the conventional winter wheat–summer corn double-cropping system, three alternative two-year, three-harvest cropping patterns—winter wheat–summer corn–spring soybean, winter wheat–summer corn–spring peanut, and winter wheat–summer corn–spring corn—significantly reduced *A. tauschii* densities. These alternative systems introduced different crops into the rotation, thereby altering the soil environment and disrupting the weed’s life cycle. This not only reduced *A. tauschii* populations but also altered the field population structure, achieving more effective overall control.

The depth of plowing is another critical factor influencing weed emergence, establishment, and persistence [37,38,39]. Environmental conditions, such as temperature, humidity, and light availability, vary with soil depth and significantly impact seedling emergence rates. When weed seeds are buried in deeper soil layers, their nutrient reserves may be depleted before they can penetrate the soil surface, leading to germination failure [40]. Laboratory experiments conducted during this study revealed that the optimal depth for *A. tauschii* seedling emergence is 1–5 cm. Emergence rates declined sharply when the depth exceeded 5 cm, and no seedlings were observed to emerge from depths greater than 15 cm. In China, shallow rotary tillage remains the predominant plowing method in agricultural systems. This practice buries *A. tauschii* seeds in shallow soil layers, creating conditions favorable for their emergence. In contrast, deep plowing, which generally reaches depths of 50–60 cm, buries weed seeds at much greater depths, where emergence is unlikely. Field experiments demonstrated that transitioning from shallow rotary tillage to deep plowing significantly reduced *A. tauschii* occurrence. This approach not only eliminated a substantial portion of the existing seed bank but also disrupted the weed’s reproductive cycle, offering a promising agronomic strategy for long-term weed management.

Water availability plays a fundamental role in seed emergence, as it activates critical biological processes, enhances enzyme activity, supplies essential nutrients for seedling growth, regulates temperature, facilitates gas exchange, and promotes early germination [41]. Controlled watering can be utilized strategically to stimulate the early germination of weed seeds, allowing for their subsequent removal through physical or chemical methods. This technique reduces the potential for future weed emergence and minimizes the competitive pressure on crops. This study clarified the role of timely watering in promoting the germination of *A. tauschii* seeds in the soil. When combined with rotary tillage during sowing, the germinated seedlings can be incorporated into the soil, where they decompose, effectively reducing *A. tauschii* populations.

Winter wheat, the predominant wheat type grown in China, often requires watering before or after sowing to ensure proper germination. Before sowing, low soil-moisture levels generally inhibit *A. tauschii* germination. However, watering during the wheat sowing period triggers the germination of these weed seeds. Wheat seedlings typically emerge within seven days of sowing, while *A. tauschii* emergence continues over a more extended period, spanning from the time of watering until the onset of winter. Delaying wheat sowing narrows the window for *A. tauschii* emergence, effectively reducing the associated weed-population size. However, it is essential to avoid excessive delays in sowing, as this could negatively impact wheat yields. Field experiments conducted during this study demonstrated the effectiveness of delayed sowing as a control measure. The results showed that appropriate delays significantly reduced *A. tauschii* emergence, with later sowing dates producing the most favorable outcomes.

## 4. Materials and Methods

### 4.1. A. tauschii Seed Dormancy Characterization

Mature *A. tauschii* seeds were collected and stored at a room temperature of 25–30 °C. Randomly selected seeds were sown weekly, and, after 14 days, the number of seedlings was recorded to calculate the emergence rate. For cultivation, nutrient soil was placed in a 7 cm diameter plastic culture basin. The soil used was garden organic nutritious soil (Xiao Tie Agriculture Industrial Park of Beijing Agricultural College, Beijing, China), with N, P, and K content ≥ 6%, organic matter ≥ 90%, and a PH value ranging from 5.5 to 6.5. The soil was thoroughly wetted by water infiltration from the bottom, after which seeds were sown at a rate of 20 spikelets per pot with six replicates. The seeds were covered with 1 cm of soil and were incubated in a light-controlled chamber (temperature: 20 °C; 12 h light/dark cycle).

Emergence percentage (%) = seedlings number/spikelets number × 100%

### 4.2. Evaluation of the Impact of Soil Depth on A. tauschii Seedling Emergence

*A. tauschii* seeds were sown in custom-made seedling tubes with a height of 50 cm. A base layer of soil was added, followed by sowing 20 spikelets and covering them with soil at depths of 0, 1, 3, 5, 7, 10, 15, 20, and 30 cm. The total soil depth was maintained at 45 cm. Each treatment was repeated four times, and the cultivation conditions were consistent with those detailed in Section 4.1.

### 4.3. Evaluation of the Impact of Environmental Factors on A. tauschii Seed Emergence

#### 4.3.1. Main Reagents

These were NaClO (Tianjin Fuyu Fine Chemical Co., Ltd., Tianjin, China), HCl (Purple Ocean Chemical Factory, Langfang, China), NaOH (Tianjin Fengchuan Chemical Reagent Co., Ltd., Tianjin, China), NaCl (Tianjin Kemiou Chemical Reagent Co., Ltd., Tianjin, China), Na_2_SO_4_ (Tianjin Fengchuan Chemical Reagent Co., Ltd., Tianjin, China), and PEG-6000 (Polyethylene glycol 6000, Tianjin Fengchuan Chemical Reagent Co., Ltd., Tianjin, China).

#### 4.3.2. Main Equipment

This comprised a PHS-3C pH meter (INASE Scientific Instrument Co., Ltd., Shanghai, China) and a MGC-250BP-2-type Illumination incubator (Shanghai Yiheng Scientific Instrument Co., Ltd., Shanghai, China).

#### 4.3.3. The Impact of Temperature Tests

A. Setting of temperature gradient

There were 6 MGC-250BP-2 illuminated incubators which were set to six temperature gradients: 5 °C, 10 °C, 15 °C, 20 °C, 25 °C, and 30 °C, respectively. The illumination was set to a 12 h light/dark cycle;

B. Seed processing and cultivation

*A. tauschii* seeds were immersed in 0.5% NaClO for 30 s, rinsed three times with distilled water, and evenly placed in 9 cm Petri dishes lined with two layers of filter paper. Each dish received 9 mL distilled water to ensure complete wetting. Twenty spikelets were used per dish, with four replicates per treatment. The Petri dishes were changed every 48 h.

#### 4.3.4. The Impact of pH, Salt Concentration, and Osmotic Potential Tests

A. Reagent preparation

a. Solutions of HCl and NaOH with different pH levels

After preparing 1 mol/L HCl and NaOH solutions, their pH levels were adjusted to 4, 5, 6, 7, 8, 9, and 10 using distilled water;

b. Solutions with different NaCl concentrations

After dissolving 4.699 g NaCl in 100 mL of distilled water, the volume was adjusted to 250 mL to produce 320 mmol/L NaCl. Then, the solution was serially diluted by half to produce 20, 40, 80, and 160 mmol/L NaCl solutions;

c. Solutions with different Na_2_SO_4_ concentrations

After dissolving 11.420 g Na_2_SO_4_ in 100 mL of distilled water, the volume was adjusted to 250 mL to produce 320 mmol/L Na_2_SO_4_. Then, the solution was serially diluted by half to produce 20, 40, 80, and 160 mmol/L solutions;

d. Solutions with different osmotic potential levels

After dissolving 18.128 g, 28.095 g, 36.323 g, 43.103 g, 49.098 g, or 54.533 g of PEG 6000 in 250 mL of distilled water, solutions with osmotic potentials of −0.1, −0.2, −0.3, −0.4, −0.5, and −0.6 MPa were prepared.

B. Seed processing and cultivation

The seeds and Petri dishes were treated as described in Section 4.3.3, except each dish received 9 mL of the test solution. The Petri dishes were incubated under standard conditions (20 °C, 12 h light/dark cycle).

#### 4.3.5. Investigation

Analyses were conducted every 24 h, recording the number of spikelets with radicle or seedling emergence. The radicle emergence criterion was a radicle length > 0.5 cm. The seedling emergence criterion was seedling length > 0.5 cm. This analysis was performed for 14 days.

$$The\;radicle\;emergence\;rate\left(\%\right)=\frac{number\;of\;spikelets\;with\;radicle}{number\;of\;tested\;spikelets}\times 100\%$$

$$The\;seedling\;emergence\;rate\left(\%\right)=\frac{number\;of\;seedlings}{number\;of\;tested\;spikelets}\times 100\%$$

### 4.4. Population Growth Dynamics of A. tauschii

Field plot experiments were conducted on the farm of the Plant Protection Institute, Hebei Academy of Agriculture and Forestry Sciences (115°31′39.52″ E, 38°51′51.85″ N) from October 2022 to July 2024. Each plot was 1 m^2^. The wheat sowing dates were 8 October 2022 and 8 October 2023, which were suitable for the sowing in terms of local production. *A. tauschii* was inoculated after wheat sowing at densities of 0, 1, 2, 4, 10, and 60 plants/m^2^ on 8 October 2022 without prior infestations. The winter wheat–summer corn double cropping system was adopted. Before wheat sowing, 60 g/m^2^ of compound fertilizer (Shandong Enbao Biological Technology Co., Ltd., Qingdao, China, N + P_2_O_5_ + K_2_O ≥ 51%; N:P:K = 18:25:8) was applied, followed by manual soil tillage, ditch digging, and seeding wheat at a rate of 22.5 g/m^2^. Watering was performed after sowing. After weeds’ head sprouting, each plot was isolated with gauze nets to prevent weed seeds from falling outside the plots. Upon wheat maturation, the wheat ears were harvested. The wheat and *A. tauschii* straws were crushed and returned to the field before the corn was seeded. There was no abnormal weather that affected the normal growth of the crops during the experiment. The density of *A. tauschii* was measured before the weeds tillering, before overwintering, and after regrowth for each growing cycle. Data collected over three years defined the population growth dynamics of *A. tauschii* and its impact on wheat.

### 4.5. Wheat Yield Loss Calculations

Based on the data from Section 4.4, after harvest, the wheat yield parameters, including the spike number, grains per spike, 1000-grain weight, and total yield, were assessed. A yield loss model was developed, correlating *A. tauschii* density after emergence with wheat yield in each growing cycle.

### 4.6. Screening for Herbicides to Control A. tauschii

The experimental field was located in Dayang Village, Lianchi District, Baoding City, Hebei Province, China (115°32′55.78″ E, 38°50′47.44″ N). This farmland, similar to the surrounding districts, followed a winter wheat–summer corn double-cropping system. Conventionally, wheat was sown around 8 October every year. After the harvest of corn and before the sowing of wheat, the land was rotary tilled, followed by seeding and fertilization using a seeding machine. The standard fertilization rate was 600 kg/hm^2^, and the wheat-seeding rate was 225 kg/hm^2^. Irrigations were carried out after wheat sowing, before overwintering, after turning green, and during the wheat-grain filling stage. Winter wheat was harvested around 10 June. Generally, summer corn was sown on the same day of wheat harvest without tillage, followed by irrigation. Summer corn was harvested around 1 October. Besides *A. tauschii*, there were *Descurainia sophia* and a small amount of *Capsella bursa-pastoris* in the wheat field, which were manually pulled out in the experiment. *A. tauschii* had the highest occurrence and was evenly distributed.

Field plot experiments (30 m^2^ per plot) were conducted to evaluate the control effects of several herbicides, including 30 g/L mesosulfuron-methyl OD (Shi ma, Bayer Aktiengesellschaft, Beijing, China), 4% pyroxsulam OD (You xian, Corteva Agriscience, Beijing, China), 70% flucarbazone-sodium WG (Biao hu, Arysta Lifescience North America, Ltd., Shanghai, China), 69 g/L fenoxaprop-p-ethyl EW (Biao ma, Bayer CropScience China Co., Ltd., Hangzhou, China), and 15% clodinafop-propargyl WP (Mai ji, Syngenta, Shanghai, China). A control treatment without herbicide application was included. The herbicides were sprayed 30 days after wheat sowing when the weeds had largely emerged. The sprayer was Knapsack Sprayer Jacto PJB-16 (INTERMAN Corporration Ltd., Rayong, Thailand) with Fan nozzle DEF-06. The spray volume of water was 450 L/hm^2^. The control effects on *A. tauschii* fresh weight were assessed the following spring using random sampling with three sampling points per plot. There was no abnormal weather that affected the experiment.

### 4.7. Control Effects of Different Cultural Methods on A. tauschii

The location of the experimental field and the conventional wheat cultivation methods were the same as described in Section 4.6.

A. Crop rotation

Field experiments tested the control efficacy associated with three two-year, three-crop rotation systems (winter wheat–summer corn–spring bean, winter wheat–summer corn–spring peanut, and winter wheat–summer corn–spring corn) compared to the conventional winter wheat–summer corn two-crop system. Each treatment had a planting area of 1500 m^2^. For the conventional system, rotary tillage, wheat seeding, fertilization and watering were performed on 9 October 2022. The winter wheat was harvested on 10 June 2023. The following summer corn was sowed on 10 June 2023, and harvested on 4 October 2023. For the three types of two-year, three-crop rotation systems, no seeding was performed after the harvest of summer corn on 9 October 2022. In the second year, watering was performed in the spring, followed by rotary tillage and land preparation. The spring-sown crops were seeded on 25 April 2023 using a seeding machine for both seeding and fertilization and they were harvested on 20 September 2023. A new wheat-growing cycle continued from 8 October 2023 to 12 June 2024, in all the four rotation systems, where *A. tauschii* density was assessed at nine sampling points before overwintering and after regrowth;

B. Deep plowing

A field experiment was conducted with treatments set at 1000 m^2^ each to assess the effect of plowing depth on controlling *A. tauschii*. Plowing depths of 5, 10, 15, 20, or 30 cm were evaluated. A rotary tilled field served as the control. Weed samples were taken from nine points in each treatment plot to record the number of *A. tauschii* plants in wheat fields before winter and after the wheat turned green;

C. Watering-induced emergence

A field experiment was carried out in a winter wheat–summer corn rotation system to test the watering-induced emergence on *A. tauschii*. Corn fields were irrigated 5, 10, 15, 20, and 25 days prior to wheat sowing on 8 October 2023. A non-irrigated field served as the control. After the corn harvest, the soil was tilled before sowing wheat. Samples were collected from nine points per treatment plot to record *A. tauschii* density in the field before sowing, before winter, and after the wheat turned green.

D. Delayed sowing

Field experiments were conducted to study the effect of delayed sowing on *A. tauschii*. Taking time as a variable, the sowing was divided into five treatments: conventional sowing on 8 October 2023 and sowing delayed by 5, 10, 15, and 20 days, each covering an area of 1000 m^2^. Samples were collected from nine points per treatment to count *A. tauschii* plants under different sowing schedules.

### 4.8. Statistical Analysis

Statistical analysis was performed using SPSS software version 21.0. The characterization of *A. tauschii* seed dormancy and screening for herbicides to control *A. tauschii* used one-way analysis of variance (ANOVA), and the mean values of each treatment group were compared using Duncan’s test at *p* < 0.05. Other experiments employed two-way ANOVA, incorporating main effect terms and their interaction term in the model. If the interaction effect was significant, further analysis was conducted through Simple Effects Analysis to clarify differences among various level combinations. If the main effect was significant but there was no interaction, Duncan’s multiple range test was used for pairwise comparisons between groups, with a significance level set at *p* < 0.05.

## 5. Conclusions

*Aegilops tauschii*, one of the most harmful and invasive weeds in wheat fields, poses a significant threat to wheat yields and, in extreme cases, can even lead to complete crop failure. Controlling *A. tauschii* is critical for ensuring food-production security. This weed is characterized by remarkable fecundity, adaptability, and self-protection mechanisms. It aligns its germination period with the wheat-sowing season, taking advantage of shallow rotary tillage to establish favorable conditions for emergence. Once established, *A. tauschii* exhibits strong colonization capabilities, rapidly expanding its population. While its initial impact may appear minimal, the subsequent effects on crop yields can be severe and widespread. Currently, mesosulfuron-methyl is the only herbicide proven effective against *A. tauschii* in wheat fields. However, relying solely on chemical herbicides is not sustainable. Instead, the integration of agronomic practices, such as crop rotation, deep plowing, controlled watering, and delayed sowing, offers a more comprehensive approach to managing *A. tauschii*. This study evaluated the efficacy of various cultural control methods, providing valuable insights into sustainable weed-management strategies. These findings are significant as they contribute to ensuring food security, enhancing agricultural production efficiency, and supporting sustainable farming practices. By adopting these strategies, farmers can mitigate the harmful effects of *A. tauschii*, promoting the health and resilience of wheat-production systems.

## Data Availability

No new data were created or analyzed in this study. Data sharing is not applicable to this article.
